# Clinicopathological Correlation in Asian Patients with Biopsy-Proven Lupus Nephritis

**DOI:** 10.1155/2015/857316

**Published:** 2015-03-19

**Authors:** Bancha Satirapoj, Pamila Tasanavipas, Ouppatham Supasyndh

**Affiliations:** Division of Nephrology, Department of Medicine, Phramongkutklao Hospital and College of Medicine, Bangkok 10400, Thailand

## Abstract

A total of 244 patients with lupus nephritis (219 women (89.8%) with a female to male ratio of 9 : 1) were included in the study. Clinical and laboratory findings at renal biopsy are clinically valuable in identifying different renal classifications of lupus pathology, activity, and chronicity index. Patients with class IVG had significantly higher proportions of microscopic hematuria, proteinuria, hypertension, impaired renal function, anemia, hypoalbuminuria, and positive anti-DNA antibody. All of these findings correlated well with high activity index and chronicity index of lupus pathology. Considering these correlations may help to determine the clinicopathologic status of lupus patients.

## 1. Introduction

Renal involvement is one of the most severe complications of systemic lupus erythematosus (SLE) and the clinical presentation of lupus nephritis (LN) is highly variable, ranging from mild asymptomatic proteinuria to rapidly progressive glomerulonephritis [1, 2]. The renal morphological expression can vary considerably among patients or within an individual over time [3, 4]. Performing renal biopsies to accurately determine the prognosis and to guide treatment in LN patients is greatly needed. Recently, the International Society of Nephrology/Renal Pathology Society (ISN/RPS) 2003 classification of LN was proposed [5].

It has been generally agreed upon that renal morphological lesions and some clinical and laboratory features are correlated. Several studies are available on the clinicopathological correlation in LN subjects mainly from developed countries [6, 7]. However, very few publications are currently available concerning the demographic, clinical, and pathological features of LN and their correlations in Thailand [8]. Therefore, this study aimed to assess the clinical and basic laboratory features of Thai patients with biopsy-proven LN class according to ISN/RPS 2003 classification, renal pathological activity, and chronicity index in patients with LN. Clinical records were reviewed and information regarding renal function, serological activity, and urinary protein is collected and compared across histological classes.

## 2. Methods

### 2.1. Subjects

Patients with LN who underwent renal biopsy between 2010 and 2013 in Phramongkutklao and Siriraj Hospitals, Bangkok, Thailand, were included in this study. All patients met the American College of Rheumatology (ACR) revised criteria for the classification of SLE [9]. For inclusion, patients had to have adequate renal biopsy samples for histological diagnosis, including >10 glomeruli. For the patients who had repeated biopsies, samples and clinical records at the time of the first biopsy were used. Consequently, data of 244 patients were available in the study. For included patients, clinical records and laboratory parameters at the time of biopsy were collected. The research followed the tenets of the Declaration of Helsinki; informed consent was obtained, and the research was approved by the institutional review board.

### 2.2. Renal Pathological Diagnosis

Renal biopsy-confirmed LN cases were classified according to the 2003 ISN/RPS classification [5]. Data regarding immunofluorescence findings were available for 100% of the patients. Activity indices (AIs) and chronicity indices (CIs) were calculated (maximum scores, 24 for AI and 12 for CI) and interstitial fibrosis was evaluated for each biopsy specimen and was graded semiquantitatively using a scoring system from 0 to 3 (0 = no changes, 1 = mild, 2 = moderate, and 3 = severe) [10].

### 2.3. Data Collection

Demographic data (sex, age, and duration of disease), extrarenal SLE manifestations, systolic blood pressure (SBP), diastolic blood pressure (DBP), anti-dsDNA antibody, anti-nuclear antibodies, and biochemical parameters white blood cells, hemoglobin, blood urea nitrogen, serum creatinine and estimated glomerular filtration rate (GFR), serum albumin, serum complement, urine protein creatinine ratio (UPCR), and previous immunosuppressive treatments at the time of kidney biopsy were obtained from the patient records. Nephrotic syndrome was defined as clinical edema, an increase in the UPCR of ≥3.5 g/gCr and serum cholesterol of >200 mg/dL, and a decrease in serum albumin to ≤3.0 g/dL. Nephrotic range proteinuria was defined as UPCR of ≥3.5 g/gCr.

### 2.4. Statistical Analysis

Data with normal distribution and nonnormal distribution were summarized as mean ± SD and median and range, respectively. Categorical variables were presented as percentage. For multiple comparisons, univariate analysis of variance (ANOVA) with post hoc analysis by Dunnett *t* was used followed by the least significance difference test. Pearson's correlation and Chi square tests were used to compare frequency variables and correlation among different variables. Multivariate model using a stepwise regression analysis was performed to correct for confounders. A backward stepwise elimination process was used to remove any other covariates. All statistical evaluations were performed using the SPSS Program, version 12.0 (SPSS, Chicago, IL, USA). A *P* value of <0.05 was considered statistically significant.

## 3. Results

A total of 244 patients were included in this study. The mean patient age at renal biopsy was 34.0 ± 12.0 years. Significant differences of mean age were observed between LN classes and in particular mean age was significantly low in patients with class IVG compared with patients with class V. Of the 244 patients, 219 (89.8%) were female with a female to male ratio of 9 : 1. Various treatment regimens were used, all of which included prednisolone (92.6%), cyclophosphamide (34.8%), azathioprine (29.5%), and mycophenolate mofetil (MMF) (16.0%) (Table 1).

### 3.1. Clinical and Laboratory Parameters Associated with Each LN Class (Tables 1 and 2)

In patients with LN class II, blood pressure was well controlled and renal function was well preserved: mean serum creatinine was 1.2 ± 0.8 mg/dL and mean estimated GFR was 76 ± 32.4 mL/min/1.73 m^2^. Urinary findings were microscopic hematuria (57.1%) and mild degree of proteinuria with mean urinary protein of 1.3 ± 0.5 g/gCr. Importantly, no patients with nephrotic syndrome or nephrotic range protein were detected. Patients with serologically active anti-dsDNA antibody comprised 42.9%.

Data were compared between class III and other classes. In class III, blood pressure was well controlled. Renal function was well preserved and no significant difference was observed with classes II and IV: mean serum creatinine was 0.9 ± 0.5 mg/dL and mean estimated GFR was 98.2 ± 31.7 mL/min/1.73 m^2^. Microscopic hematuria was significantly higher in patients with class III (63.2%) than in patients with class V (32.1%). Urinary protein excretion was moderate: mean urinary protein was 3.4 ± 2.4 g/gCr, and frequency of nephrotic syndrome was 18.4%. Significant differences were found in levels of proteinuria in class III and class II. Serologically active anti-dsDNA antibody (86.8%) in patients with class III was significantly higher than in patients with class II (42.9%).

Patients with class IVG had the most severe nephritis as follows: SBP, 144.2 ± 22.8 mmHg; DBP, 89.5 ± 14.2 mmHg; serum creatinine, 1.5 ± 1.1 mg/dL; estimated GFR, 68.4 ± 37.2 mL/min/1.73 m^2^; urinary protein, 4.7 ± 3.3 g/gCr; serum albumin, 2.8 ± 0.6 g/dL; and hemoglobin, 10 ± 2.2 g/dL. At the time of renal biopsy, 92 (78.6%) patients were hypertensive (blood pressure ≥ 140/90 mmHg) and 44 (37.6%) patients exhibited impaired GFR (GFR < 50 mL/min/1.73 m^2^). In comparison with other classes, patients with class IVG had a significant increase in serum creatinine, blood pressure, proteinuria, and frequency of microscopic hematuria and a significant decrease in estimated GFR, serum albumin, and hemoglobin (Tables 1 and 2). A significantly high frequency of nephrotic syndrome (33.3%) and active anti-dsDNA antibody (80.7%) were observed. Data were similar between classes IVG and IVS, but patients with class IVS tended be less severe nephritis profiles including blood pressure, renal function, serum albumin, hemoglobin, and frequency of nephrotic syndrome.

In class V, blood pressure was well controlled. Renal function was significantly higher in class V patients than patients with class IVG (estimated GFR 95.3 ± 38.2 versus 68.4 ± 37.2 mL/min/1.73 m^2^, *P* < 0.05). Microscopic hematuria was significantly lower in patients with class V (32.6%) than in patients with class IVG (73.5%) and class IVS (74.4%). Urinary protein excretion was high: mean urinary protein was 5.1 ± 3.8 g/gCr, and a significantly high frequency of nephrotic syndrome (41.9%) was observed compared with class II. However, no significant differences were found in levels of proteinuria in class V with class IV. Serologically active anti-dsDNA antibody in patients with class V was 69.8%.

### 3.2. Renal Pathological Findings

The relative frequency of each class was as follows: class II (mesangial proliferative LN) 2.9%, class III (focal proliferative LN) 15.6%, class IVG (diffuse global proliferative LN) 50%, class IVS (diffuse segmental proliferative LN) 16%, and class V (membranous LN) 17.6%. Patients of class III + V were considered to be class III and patients of class IV + V were considered to be class IV. No class VI was diagnosed at the initial renal biopsy. Patients with classes III and IV had a higher activity index, percentage of endocapillary proliferation, glomerular leukocyte infiltration, hyaline thrombi, fibrinoid necrosis, cellular crescents, and interstitial inflammation (Table 3). Patients with class IV had a higher chronicity index, percentage of fibrous crescents, tubular atrophy, and interstitial fibrosis (Table 3).

### 3.3. Clinical Parameters Associated with Renal Pathological Activity and Chronicity Index

Univariate analysis was performed to assess the relationship between clinical parameters with renal pathological activity and chronicity index. SBP, DBP, BUN, serum creatinine, UPCR, and presence of serositis, microscopic hematuria, and anti-dsDNA were positively correlated with renal pathological activity index, whereas age, estimated GFR, hemoglobin, and serum albumin were negatively correlated with renal pathological activity index. After multiple regression analyses, significant correlations were found among age, DBP, serum creatinine, estimated GFR, hemoglobin, serum albumin, UPCR, and presence of microscopic hematuria and anti-dsDNA antibody with renal pathological activity index (Table 4).

Renal pathological chronicity index was positively correlated significantly with SBP, DBP, BUN, serum creatinine, UPCR, and presence of serositis but negatively correlated with estimated GFR, hemoglobin, and serum albumin. After multiple regression analyses, significant correlations were found among serum creatinine, estimated GFR, hemoglobin, UPCR, and presence of microscopic hematuria and anti-dsDNA antibody with renal pathological chronicity index (Table 5).

## 4. Discussion

The study demonstrated the correlation of various demographic and laboratory findings in cases of biopsy-proven LN with the pathological features on renal biopsies. One of the most common findings was class IVG (48%). At renal biopsy, a higher microscopic hematuria, impaired GFR, proteinuria, anemia, hypoalbuminemia and hypertension, and the presence of positive anti-DNA antibody were all associated with the worst class, that is, class IV. These parameters were also correlated with high renal pathological activity and chronicity index in patients with LN.

Renal survival is mainly determined by the severity of renal involvement and renal classification [11]. The discrepancy may be related to differences in ethnicity or to different severity of renal disease in LN at biopsy. Several clinical characteristics related to lupus classification in our study confirmed those previously reported [12–14]. Initially, significant differences were observed in the degree of nephritic and nephrotic features including microscopic hematuria, high blood pressure, impaired renal function, high proteinuria, and presence of anti-dsDNA antibody between LN classes II and IVG/IVS. Importantly, no patients with nephrotic syndrome or nephrotic range proteinuria were detected in class II. Unfortunately, the data in our series offered no way of distinguishing among the different clinical findings among patients with LN classes III and IV. Additionally, there was no difference in all extrarenal manifestations among each class.

Our patients with class IV or V had massive proteinuria with lower serum albumin levels than those of patients with class II. Regarding the difference between class IV and class V, no statistical difference was found in proteinuria, serum albumin, and cholesterol levels in this study, suggesting that all clinical features of nephrotic syndrome may be unimportant to define LN class IV and class V, whereas significant differences were observed in nephritic parameters including microscopic hematuria, anemia, high blood pressure, and impaired renal function between LN classes V and IV in initial biopsy finding. The results from our study on biopsy-proven cases of LN largely concur with the previously reported study [15]. Finally, our findings indicated that, at the same level of proteinuria without microscopic hematuria, anemia, high blood pressure, and impaired renal function might prefer diagnosis of LN class V compared with LN class IV.

Histological activity and chronicity index were strongly predictive for renal function outcome [16] and these measurements were used to grade the individual morphologic components in a given biopsy as a guide to treat and predict the renal survival in patients with LN [17, 18]. Previous studies have shown that serum creatinine and proteinuria had a significant positive correlation with high activity and chronicity index on pathological features of LN [13, 14]. Our results support this finding; advanced age and nephritic parameters including blood pressure, serum creatinine, estimated GFR, hemoglobin, serum albumin, UPCR, and presence of microscopic hematuria and anti-dsDNA antibody had a significant positive correlation with high renal pathological activity and chronicity index. We speculated that all nephritic features might reflect renal activity and chronicity scores, especially immunologic disease activities in patients with SLE.

Our study had several limitations, including its retrospective design and assessment of a small number of patients in LN class II. Furthermore, a possibility of differences in immunosuppressive agents may have occurred before inclusion and during the follow-up period. Evaluation of other clinical factors potentially affecting clinical features, such as other medications and socioeconomic status, would be interesting but was undetermined in this study. Larger prospective trials are still required to ascertain consistent baseline clinical markers.

In conclusion, this study indicates that clinical and laboratory findings at renal biopsy are clinically valuable in identifying different renal classifications of lupus pathology, activity, and chronicity index. Our results suggest that patients with class IVG had significantly higher proportions of microscopic hematuria, proteinuria, hypertension, impaired renal function, anemia, hypoalbuminuria, and positive anti-DNA antibody. All of these findings correlated well with high activity index and chronicity index of lupus pathology.

## Figures and Tables

**Table 1:** The demographic and clinical findings at renal biopsy by LN class.

	Total (*N* = 244)	Class II (*N* = 7)	Class III (*N* = 38)	Class IVG (*N* = 117)	Class IVS (*N* = 39)	Class V (*N* = 43)
Female (*n*, %)	219 (89.8%)	5 (71.4%)	35 (92.1%)	106 (90.6%)	34 (87.2%)	39 (90.7%)
Age (years)	34 ± 12	42 ± 9.8	34.7 ± 13.2	32 ± 11.4^†^	33 ± 11.1	38.6 ± 12.5
Current immunosuppressive agents (*n*, %)						
Prednisolone	226 (92.6%)	6 (85.7%)	37 (97.4%)	104 (88.9%)	38 (97.4%)	41 (95.3%)
Azathioprine	72 (29.5%)	2 (28.6%)	9 (23.7%)	31 (26.5%)	14 (35.9%)	16 (37.2%)
Cyclophosphamide	85 (34.8%)	2 (28.6%)	14 (36.8%)	40 (34.2%)	13 (33.3%)	16 (37.2%)
Mycophenolate mofetil	39 (16%)	1 (14.3%)	8 (21.1%)	19 (16.2%)	7 (17.9%)	4 (9.3%)
Current antihypertensive agents (*n*, %)						
ACEI/ARB	102 (41.8%)	4 (57.1%)	12 (31.6%)	45 (38.5%)	19 (48.7%)	22 (51.2%)
CCB	59 (24.2%)	3 (42.9%)	3 (7.9%)^*^	36 (30.8%)	9 (23.1%)	8 (18.6%)
BB	15 (6.1%)	—	—	8 (6.8%)	5 (12.8%)	2 (4.7%)
Extrarenal manifestation (*n*, %)						
Arthritis	134 (54.9%)	2 (28.6%)	28 (73.7%)	59 (50.4%)	20 (51.3%)	25 (58.1%)
Malar rash	112 (45.9%)	2 (28.6%)	16 (42.1%)	57 (48.7%)	20 (51.3%)	17 (39.5%)
Discoid rash	60 (24.6%)	1 (14.3%)	6 (15.8%)	28 (23.9%)	10 (25.6%)	15 (34.9%)
Photosensitivity rash	45 (18.4%)	1 (14.3%)	7 (18.4%)	20 (17.1%)	8 (20.5%)	9 (20.9%)
Oral ulcer	66 (27%)	—	11 (28.9%)	39 (33.3%)	6 (15.4%)	10 (23.3%)
AIHA	79 (32.4%)	—	13 (34.2%)	45 (38.5%)	10 (25.6%)	11 (25.6%)
Leukopenia	86 (35.2%)	1 (14.3%)	18 (47.4%)	44 (37.6%)	11 (28.2%)	12 (27.9%)
Thrombocytopenia	64 (26.2%)	1 (14.3%)	8 (21.1%)	30 (25.6%)	14 (35.9%)	11 (25.6%)
Serositis	50 (20.5%)	—	6 (15.8%)	32 (27.6%)	8 (20.5%)	4 (9.3%)
CNS	11 (4.5%)	—	—	7 (6%)	—	4 (9.3%)

Data are mean ± SD, median with interquartile range and percentages; ^†^ *P* < 0.05 versus class V and ^*^ *P* < 0.05 versus class II. ACEI: angiotensin converting enzyme inhibitor, AIHA: autoimmune hemolytic anemia, ARB: angiotensin type 1 receptor blocker, BB: beta-blockers, CCB: calcium channel blocker, CNS: central nervous system.

**Table 2:** Renal findings at renal biopsy by LN class.

	Total (*N* = 244)	Class II (*N* = 7)	Class III (*N* = 38)	Class IVG (*N* = 117)	Class IVS (*N* = 39)	Class V (*N* = 43)
Levels of clinical and laboratory findings						
SBP (mmHg)	138.9 ± 22.7	118.4 ± 14.7	131.1 ± 17.9	144.2 ± 22.8^*^	139.3 ± 22.3^*^	134.6 ± 23.9
DBP (mmHg)	85.8 ± 14.4	76.1 ± 10.7	81 ± 12.1	89.5 ± 14.2^†∗^	86.1 ± 14.8	81.1 ± 13.9
Hemoglobin (g/dL)	10.7 ± 2.2	12.5 ± 1.3	11.2 ± 2.2	10 ± 2.2^†∗^	10.7 ± 1.5^†^	12 ± 1.9
BUN (mg/dL)	27.9 ± 21.4	14.7 ± 4.7	18.4 ± 12.3	33.5 ± 24.3^†∗^	28.8 ± 17.4	22.2 ± 19.6
Serum creatinine (mg/dL)	1.3 ± 1	1.2 ± 0.8	0.9 ± 0.5	1.5 ± 1.1^†^	1.3 ± 1	1.1 ± 1.1
Estimated GFR (mL/min/1.73 m^2^)	80.2 ± 38.3	76 ± 32.4	98.2 ± 31.7	68.4 ± 37.2^†^	82 ± 36.9	95.3 ± 38.2
Serum cholesterol (mg/dL)	287.2 ± 107.6	225.7 ± 45.7	249.5 ± 74.5	296.2 ± 105.6	302 ± 138.3	297.3 ± 113.9
Serum albumin (g/dL)	2.9 ± 0.7	3.9 ± 0.5	3 ± 0.8^*^	2.8 ± 0.6^*^	2.9 ± 0.6^*^	2.9 ± 0.6^*^
UPCR (g/g creatinine)	4.4 ± 3.2	1.3 ± 0.5^†^	3.4 ± 2.4^*^	4.7 ± 3.3^*^	4.2 ± 2.4^*^	5.1 ± 3.8^*^
Percentages of clinical and laboratory findings						
Hypertension (*n*, %)	163 (66.8%)	3 (42.9%)	18 (47.4%)	92 (78.6%)^†^	26 (66.7%)	24 (55.8%)
Microscopic hematuria (*n*, %)	157 (64.3%)	4 (57.1%)	24 (63.2%)^†^	86 (73.5%)^†^	29 (74.4%)^†^	14 (32.6%)
GFR < 50 mL/min/1.73 m^2^	68 (27.9%)	2 (28.6%)	5 (13.2%)	44 (37.6%)^†^	10 (25.6%)	7 (16.3%)
Nephrotic range proteinuria (*n*, %)	125 (51.2%)	—	14 (36.8%)	67 (57.3%)^*^	19 (48.7%)^*^	25 (58.1%)^*^
Nephrotic syndrome (*n*, %)	74 (30.3%)	—	7 (18.4%)	39 (33.3%)^*^	10 (25.6%)	18 (41.9%)^*^
Anti-dsDNA (*n*, %)	190 (77.9%)	3 (42.9%)	33 (86.8%)^*^	92 (80.7%)^*^	32 (86.5%)^*^	30 (69.8%)

Data are mean ± SD, median with interquartile range and percentages; ^†^ *P* < 0.05 versus class V and ^*^ *P* < 0.05 versus class II. BUN: blood urea nitrogen, DBP: diastolic blood pressure, and GFR: glomerular filtration rate. UPCR: urine protein creatinine ratio and SBP: systolic blood pressure.

**Table 3:** The renal pathological findings at renal biopsy by LN class.

	Total (*N* = 244)	Class II (*N* = 7)	Class III (*N* = 38)	Class IVG (*N* = 117)	Class IVS (*N* = 39)	Class V (*N* = 43)
Activity index						
Endocapillary proliferation	184 (75.4%)	1 (14.3%)	34 (89.5%)^†∗^	108 (92.3%)^†∗^	39 (100%)^†∗^	2 (4.7%)
Glomerular leukocyte infiltration	148 (60.7%)	1 (14.3%)	20 (52.6%)^†∗^	92 (78.6%)^†∗^	30 (76.9%)^†∗^	5 (11.6%)
Hyaline thrombi	154 (63.1%)	0 (0%)	17 (44.7%)^†∗^	100 (85.5%)^†∗^	33 (84.6%)^†∗^	4 (9.3%)
Fibrinoid necrosis	41 (16.8%)	1 (14.3%)	6 (15.8%)	23 (19.7%)^†^	11 (28.2%)^†^	0 (0%)
Cellular crescent	94 (38.5%)	0 (0%)	15 (39.5%)^†^	58 (49.6%)^†∗^	20 (51.3%)^†∗^	1 (2.3%)
Interstitial inflammation	150 (61.5%)	3 (42.9%)	22 (57.9%)	78 (66.7%)^†^	28 (71.8%)	19 (44.2%)
Total activity index (Max 24)	6.8 ± 4.3	1.1 ± 2.2	4.4 ± 2.3^†∗^	9.4 ± 3.1^†∗^	8.6 ± 2.9^†∗^	1.2 ± 1.8
Median (IQR)	7 (3, 10)	0 (0, 1)	4 (3, 6)	9 (7, 11.5)	8 (6, 11)	1 (0, 1)
Chronicity index						
Sclerotic glomeruli	121 (49.6%)	4 (57.1%)	16 (42.1%)	55 (47%)	26 (66.7%)	20 (46.5%)
Fibrous crescent	53 (21.7%)	0 (0%)	5 (13.2%)	33 (28.2%)^†^	15 (38.5%)^†∗^	0 (0%)
Tubular atrophy	121 (49.6%)	1 (14.3%)	12 (31.6%)	64 (54.7%)	23 (59%)^*^	21 (48.8%)
Interstitial fibrosis	139 (57%)	1 (14.3%)	14 (36.8%)	73 (62.4%)^*^	27 (69.2%)^*^	24 (55.8%)
Total chronicity index (Max 12)	2.5 ± 2.3	1.1 ± 1.5	1.6 ± 1.9	2.9 ± 2.4	3 ± 2.1^*^	2.2 ± 2.3
Median (IQR)	2 (0, 4)	1 (0, 2)	1 (0, 3)	3 (1, 4)	3 (2, 4)	2 (0, 3)

Data are mean ± SD, median with interquartile range and percentages; ^†^ *P* < 0.05 versus class V and ^*^ *P* < 0.05 versus class II.

**Table 4:** Univariate and multivariate regression analyses demonstrating clinical factors showing correlation with renal pathological activity index (AI).

	Univariate regression			Multivariate regression *R* ^2^ = 0.386		
	Beta	SE.	*P* value	Beta	SE.	*P* value
Age (yrs)	−0.09	0.02	<0.001	−0.10	0.02	<0.001
SBP (mmHg)	0.05	0.01	<0.001			
DBP (mmHg)	0.08	0.02	<0.001	0.05	0.02	0.001
Serositis	1.97	0.68	0.004			
Anti-dsDNA	2.23	0.69	0.001	1.18	0.57	0.039
Blood urea nitrogen (mg/dL)	0.08	0.01	<0.001			
Serum creatinine (mg/dL)	1.43	0.27	<0.001	−0.98	0.43	0.024
Estimated GFR (mL/min/1.73 m^2^)	−0.04	0.01	<0.001	−0.05	0.01	<0.001
Hemoglobin (g/dL)	−0.81	0.12	<0.001	−0.40	0.12	0.001
Serum albumin (g/dL)	−1.21	0.40	0.003			
UPCR (g/g creatinine)	0.21	0.09	0.016	0.18	0.07	0.014
Microscopic hematuria	2.50	0.56	<0.001	1.44	0.48	0.003

Independent variables in the multivariate model were chosen using a stepwise regression analysis where all significant variables listed in the univariate analysis were included.

DBP: diastolic blood pressure, GFR: glomerular filtration rate, SBP: systolic blood pressure, and UPCR: urine protein creatinine ratio.

**Table 5:** Univariate and multivariate regression analyses demonstrating clinical factors showing correlation with renal pathological chronicity index (CI).

	Univariate regression			Multivariate regression *R* ^2^ = 0.409		
	Beta	SE.	*P* value	Beta	SE.	*P* value
Age (yrs)	0.01	0.01	0.762			
SBP (mmHg)	0.02	0.01	0.001			
DBP (mmHg)	0.03	0.01	0.006			
Serositis	0.86	0.37	0.020			
Anti-dsDNA	0.64	0.38	0.092	0.67	0.30	0.026
Blood urea nitrogen (mg/dL)	0.04	0.01	<0.001			
Serum creatinine (mg/dL)	1.17	0.13	<0.001	0.62	0.21	0.004
Estimated GFR (mL/min/1.73 m^2^)	−0.03	0.00	<0.001	−0.02	0.01	0.008
Hemoglobin (g/dL)	−0.37	0.07	<0.001	−0.20	0.06	0.002
Serum albumin (g/dL)	−0.39	0.22	0.077			
UPCR (g/g creatinine)	0.13	0.05	0.005	0.12	0.04	0.003
Microscopic hematuria	−0.58	0.31	0.063	−1.06	0.26	<0.001

Independent variables in the multivariate model were chosen using a stepwise regression analysis where all significant variables listed in the univariate analysis were included.

DBP: diastolic blood pressure, GFR: glomerular filtration rate, SBP: systolic blood pressure, and UPCR: urine protein creatinine ratio.
